# Acupuncture for premature ejaculation: Protocol for a systematic review

**DOI:** 10.1097/MD.0000000000011980

**Published:** 2018-08-21

**Authors:** Qi Zhao, Hengheng Dai, Xihao Gong, Lu Wang, Minran Cao, Haisong Li, Bin Wang

**Affiliations:** a Graduate School of Beijing University of Chinese Medicine; b Department of Andrology, Dongzhimen Hospital, Beijing, China.

**Keywords:** acupuncture, premature ejaculation, protocol, systematic review

## Abstract

**Background:**

Premature ejaculation is a common sexual dysfunction disease in adult males. It can be divided into primary and secondary premature ejaculation. Acupuncture is widely used in the treatment of premature ejaculation in China. There are many clinical trials confirmed that acupuncture can prolong the ejaculation latency in the vagina. We aim to use a meta-analysis to evaluate the efficacy and safety of acupuncture for premature ejaculation.

**Method:**

We will systematically search all randomized controlled trials (RCTs) by electronic and manual search, until June 31, 2018. Electronic retrieval of the database includes Medline, EMBASE, the Cumulative Index to Nursing and Allied Health Literature, Allied and Complementary Medicine Database, the Cochrane Library, the Chinese BioMedical Literature Database, the China National Knowledge Infrastructure (CNKI), the China Science and Technology Journal database (VIP), and the Wanfang database. Manual search will retrieve gray literature, including unpublished conference articles. The primary outcomes include the Intravaginal Ejaculatory Latency Time (IELT). At the same time, Premature Ejaculation Diagnostic Tool (PEDT), Arabic index of Premature Ejaculation (AIPE), Index of Premature Ejaculation (IPE) will be the secondary outcomes. Two reviewers will independently read the articles, extract the data information, and give the assessment of risk of bias. Data analysis will be used the special software like RevMan (version 5.3) and EndNote X7.

**Ethics and dissemination:**

This systematic review will evaluate the efficacy and safety of acupuncture for premature ejaculation. This review does not require ethical approval and will be reported in a peer-reviewed journal.

**PROSPERO registration number:**

PROSPERO CRD42018092783.

## 1 Introduction

Premature ejaculation (PE) is a common sexual dysfunction disease, about 14% -30% of adult men suffering from PE.^[1,2]^ However, the definition of PE is still unclear. According to the European Society of Surgeons (ESS), PE is defined as the Intravaginal Ejaculatory Latency Time (IELT) less than 1 minute (primary PE), or the former is shortened to less than 3 minutes (secondary PE), accompanied by the inability to delay ejaculation, and mental health problems.^[3]^ The cause of PE is still unclear. The mainstream view suggests that it may be related to serotonin neurotransmitters,^[4]^ penile head sensitivity,^[5]^ abnormal thyroid function,^[6]^ and emotional factors.^[7]^ PE has a great psychological impact on patients, leading to mental distress, anxiety, paralysis, and depression, having a huge impact on the patient's quality of life.^[8]^

Treatments for PE mainly include drug therapy^[9]^ and psychological and behavioral therapy.^[10]^ Oral 5-HT receptor reuptake inhibitors (SSRIs) are well-established and effective therapies for the treatment of PE, including fluoxetine,^[11]^ paroxetine,^[12]^ sertraline,^[13]^ dapoxetine,^[14]^ and so on. However, the side effects of SSRIs, such as nausea, vomiting, and dry mouth are somewhat confusing for clinicians.^[15]^ At the same time, evidence shows that the efficacy of psychological and behavioral therapy is also not clear.^[16,17]^

As a part of traditional Chinese medicine, acupuncture therapy has been widely used in clinical trials of PE in recent years. Recent studies have shown that acupuncture can extend the ejaculation latency to a certain extent.^[18]^ On the basis of TCM theory, acupuncture can regulate the balance of qi and blood by stimulating acupuncture points to improve human body function. Studies have shown that acupuncture tianshu (ST25), zusanli (ST36), and taichong (LR3) can adjust neurotransmitter 5-HT levels and reduce nerve sensitivity.^[19]^

In the preliminary searches of the electronic databases, we found that randomized controlled trials (RCTs) of acupuncture for PE are on the rise.^[20,21]^ However, due to the limitation of the size and number of clinical centers, most clinical trials are small samples with low-quality and lack of evidence-based exploration. Besides, the publication of the similar systematic review has not been retrieved in the database. Therefore, this review hopes to adopt meta-analysis to evaluate the efficacy and safety of acupuncture in the treatment of PE and provide evidence for its application in clinical practice.

## 2 Methods

This systematic review protocol has been registered on PROSPERO as CRD42018092783.(https://www.crd.york.ac.uk/PROSPERO/display_record.php?RecordID=92783) The protocol follows the Cochrane Handbook for Systematic Reviews of Interventions and the Preferred Reporting Items for Systematic Reviews and Meta-Analysis Protocol (PRISMA-P) statement guidelines.^[22]^ We will describe the changes in our full review if needed.

### 2.1 Inclusion criteria for study selection

#### 2.1.1 Types of studies

The type of literature included will be RCTs of acupuncture therapy for PE. The language is limited to Chinese and English. Non-RCTs, quasi-RCTs, case series, case reports, and crossover studies will be excluded.

#### 2.1.2 Types of participants

The cases included are adult male patients over 18 years old who have diagnosed PE. The region, nation, ethnic, and sources are not limited.

#### 2.1.3 Types of interventions

##### 2.1.3.1 Experimental interventions

Acupuncture therapies as experimental groups can be included, such as acupuncture, manual acupuncture, electro-acupuncture, fire needle, plum blossom needle, skin needle, and acupressure massage. Pharmaco-acupuncture and acupoint injection will be rejected, as their methods and theories are different from TCM. The treatment duration and frequency are not limited.

##### 2.1.3.2 Control interventions

The control groups can be using no treatment, sham acupuncture, and placebo acupuncture. Such RCT research will be excluded as acupuncture combined with traditional Chinese medicine, moxibustion, and other TCM treatment.

The following treatment comparisons will be investigated:

(1) Acupuncture versus no treatment;
(2) Acupuncture versus placebo/sham acupuncture;
(3) Acupuncture versus drug therapy;
(4) Acupuncture versus other active therapies;
(5) Acupuncture with another active therapy versus the same therapy alone.

#### 2.1.4 Types of outcome measures

##### 2.1.4.1 Primary outcomes

The primary outcome measurement will be IELT.

##### 2.1.4.2 Secondary outcomes

We also need to pay attention to the following outcomes: Premature Ejaculation Diagnostic Tool (PEDT), Arabic index of Premature Ejaculation (AIPE), and Index of Premature Ejaculation (IPE). More importantly, the adverse reactions of patients during medication will also be taken seriously.

### 2.2 Search methods for the identification of studies

#### 2.2.1 Electronic searches

The literature search will be conducted in both electronic and manual search, by June 31, 2018. The electronic search will consist of 6 English databases and 4 Chinese databases, such as PubMed, EMBASE, the Cumulative Index to Nursing and Allied Health Literature, Allied and Complementary Medicine Database, the Cochrane Library, the Chinese BioMedical Literature Database, the China National Knowledge Infrastructure (CNKI), the China Science and Technology Journal database (VIP), and the Wanfang database. Team members (QZ and HHD) will develop a complete systematic search strategy based on the Cochrane Handbook Guidelines and submit it to an experienced expert (HSL) to ensure the accuracy. Following the following terms will be chosen: acupuncture, manual acupuncture, electroacupuncture, fire needle, plum blossom needle, skin needle and acupressure massage, PE, and sexual dysfunction. The search term in the Chinese database is the translation of the above word. The complete PubMed search strategy is summarized in Table 1.

**Table 1:**
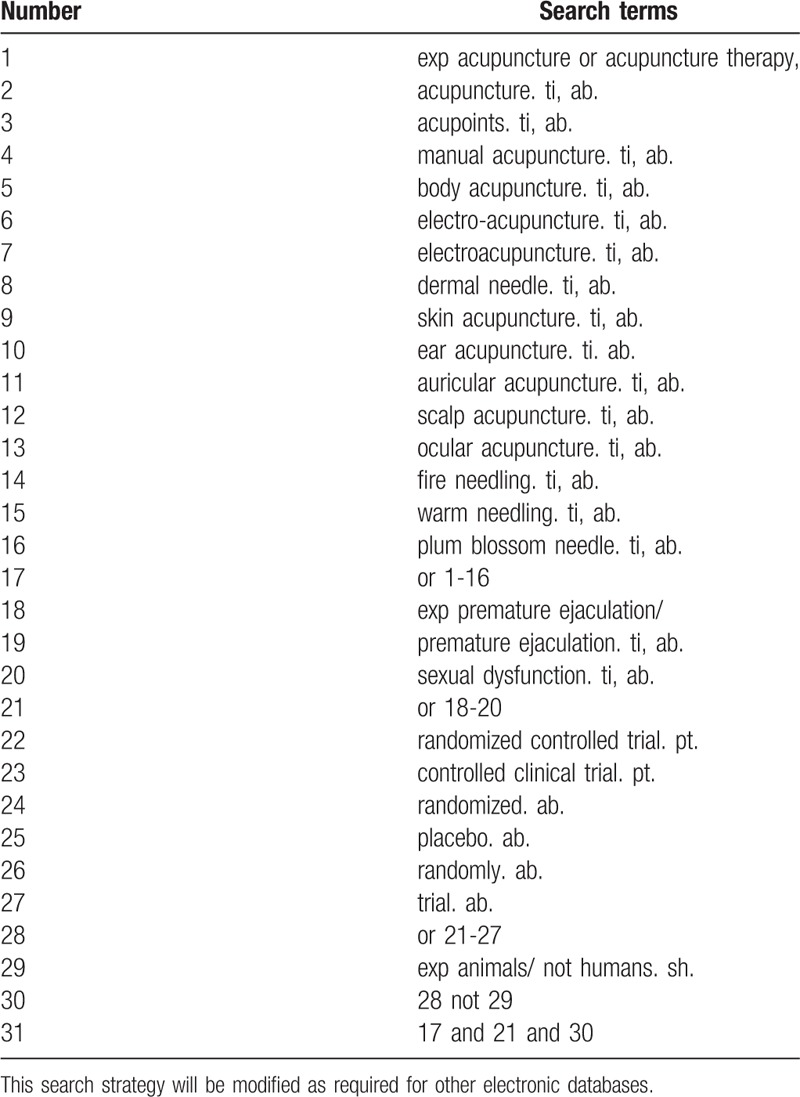
Search strategy used in PubMed database.

#### 2.2.2 Searching other resources

The manual search is mainly for dissertations, ongoing experiments, and grey literature. We will look for abstracts of dissertations, conference papers, and conference papers related to acupuncture and PE. Ongoing trials for the new reviews that are relevant to this term will be retrieved from the WHO International Clinical Trials Registry Platform (ICTRP), ClinicalTrials.gov, and the Chinese Clinical Trial Registry. For ongoing experiments, we will try to contact the trial author to help provide up-to-date clinical data. Potential gray literature will be elected in OpenGrey.eu. website.

### 2.3 Data collection and analysis

#### 2.3.1 Selection of studies

**Figure 1:**
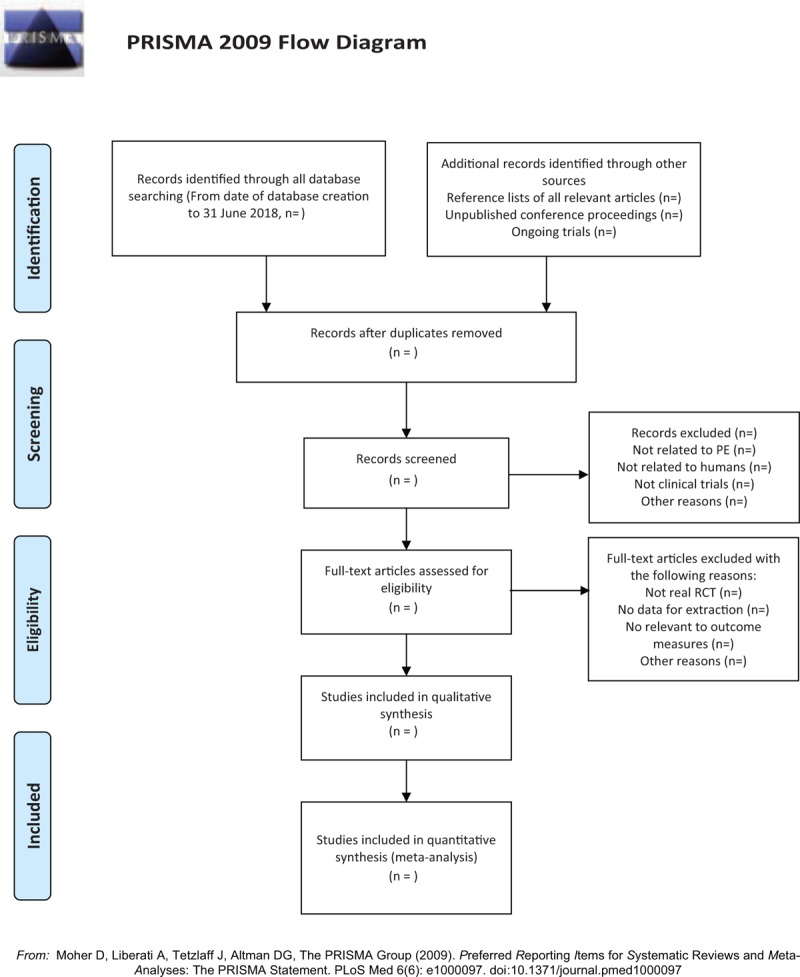
The PRISMA flowchart. *From:* Moher D, Liberati A, Tetzlaff J, Altman DG, The PRISMA Group (2009). *P*referred *R*eporting *I*tems for *S*ystematic Reviews and *M*eta-*A*nalyses: The PRISMA Statement. PLoS Med 6(6): e1000097. doi:10.1371/journal.pmed1000097.

Before the literature screening, the team members will be trained by experienced experts (BW) to ensure the risk of bias (ROB) in human factors during screening. We will use EndNote X7 literature management during the screening process. Two reviewers (QZ and HHD) read the title, abstract, and keywords and obtain the full text if necessary. The literature screening will be strictly followed the inclusion criteria. If there is disagreement during the screening process, it will be decided by another reviewer (HSL) through discussion. We will record the causes of each excluded article and submit a summary of reasons for exclusion. The details of selection process will be shown in the PRISMA flow chart (Fig. 1).

#### 2.3.2 Data extraction and management

Two reviewers (LW and MRC) will extract the necessary information for the systematic review from the documents included. This information will form a detail extraction form. If the details in the literature are incomplete, we will contact the author via email, telephone, etc. The following data will be extracted:

(1) General information: research identification, publication time, title of article, correspondent author, contact information.
(2) Study methods: study design, sample size, randomization method, allocation concealment, blinding, incomplete report or selecting report, other sources of bias.
(3) Participants: inclusion criteria and exclusion criteria, age of patients, gender, PE diagnostic criteria, severity, race, research site, baseline of ejaculation time.
(4) Interventions: type of acupuncture, needles, acupoints, the dose of the medicine, treatment duration and frequency.
(5) Outcomes: primary, secondary and safety outcomes as described above in the type of outcome measures part.
(6) Notes: financial support, conflicts of interest, ethical approval, important citations.

#### 2.3.3 Assessment of risk of bias in included studies

The quality assessment of the literature will be assessed using the ROB assessment tool in the Cochrane. Evaluation criteria include random sequence generation (selection bias), allocation concealment (selection bias), blinding of outcome assessment (detection bias), incomplete outcome data (attrition bias), selective reporting (reporting bias), and others bias. Two reviewers (XHG and LW) will evaluate each article on their own and give each index an evaluation, such as low risk, unclear, or high risk. If the result is disagreeable, it will be decided after discussion with another reviewer (BW).

#### 2.3.4 Measures of treatment effect

For continuous variable outcome, mean difference (MD) or standardized mean difference (SMD) and 95% confidence interval (95% CI) will be recorded. For dichotomous outcomes, we will use the relative risk (RR) and 95% CI records.

#### 2.3.5 Unit of analysis issue

We will only extract the first experimental period data of crossover trials to avoid carryover effects. With multiple intervention groups, we will combine all similar experimental groups and control groups into 1 group to prevent a unit of analysis issue.

#### 2.3.6 Dealing with missing data

If we find that data information is missing when we include the data, first, we will consider the reason for the information loss. After that, we will contact the author through various means, including telephone, email, etc, to supplement the lost information. If the missing information is not up-to-date, we analyze only the available data and describe it in the discussion.

#### 2.3.7 Assessment of heterogeneity

We will use complete case data as analysis data. Heterogeneity is represented with a significance level of *P* < .1 and *I*^2^ test. When *I*^2^ value ≤50% and *P* ≥ .1, the study will be understood with no statistical heterogeneity and a fixed-effect model will be used. When *I*^2^ > 50% or *P* < .1, it will be understood with significant statistical heterogeneity and a random-effect model will be used.

#### 2.3.8 Data synthesis and analysis

We will use Review Manager software (RevMan V.5.3.5) provided by Cochrane Collaboration for data synthesis and analysis. When *I*^2^ < 50%, a fixed-effects model will be used to calculate the RR and MD. When *I*^2^ ≥50%, we will use a random-effects model to synthesize the data. Subgroup analysis will be performed and the potential reasons will be analyzed to explore the causes of heterogeneity. If meta-analysis is not appropriate, we may use narrative synthesis.

#### 2.3.9 Assessment of publication bias

If 1 outcome of meta-analysis incorporates a sufficient number of articles (≥10 articles), this study will use funnel plots to visually examine the risk of publication bias. Obviously, asymmetric funnel plots indicate the risk of publication bias.

#### 2.3.10 Subgroup analysis

For the primary outcome, when there is significant heterogeneity in the meta-analysis, subgroup analysis is performed on different interventions, controls, and outcome measures.

#### 2.3.11 Sensitivity analysis

We will perform sensitivity analysis for primary outcomes to test the robustness of the review conclusions, and we will still evaluate the impact of methodological quality, sample size, and missing data.

#### 2.3.12 Grading the quality of evidence

This study will use GRADE pro online software to evaluate the included studies. The evaluation included bias risk; heterogeneity; indirectness; imprecision; publication bias. And each level of evidence will be made “very low,” “low,” “moderate,” or “high” judgment.

## 3 Discussion

In recent years, attention has been paid to the pathogenesis of neurobiology of PE, especially the 5-HT theory has become a hot topic.^[23]^ 5-HT is an important neurotransmitter, mainly distributed in the brain stem, hypothalamus, and spinal cord, and is involved in the regulation of the entire ejaculation process.^[24]^ The current study found that there are 3 subtypes involved in the regulation of animal ejaculation: among 5-HT1B and 5-HT2C receptor excitability can make IELT prolonged, which is beneficial to delay ejaculation.^[25]^ In the 1990s, Patterson reported for the first time that fluoxetine can prolong IELT. Thereafter, sertraline, paroxetine, dapoxetine, and clomipramine are widely used in the treatment of PE.^[26]^ Because of the wide distribution of 5-HT receptors, long-term use of SSRI drugs may cause dizziness, dry mouth, nausea, insomnia, fatigue, abdominal pain, diarrhea, constipation, and other adverse reactions, and in severe cases affect sexual function, loss of libido, and even sexual dysfunction.^[27,28]^

As an external treatment with low side effects and environmental protection, acupuncture has long been used to treat male diseases such as erectile dysfunction^[29]^ and chronic prostatitis.^[30]^ The way it works is mainly through promoting the operation of qi to balance the body's yin and yang.^[31]^ Although the mechanism of acupuncture treatment for PE lacks awareness, clinical studies have shown that acupuncture can prolong ejaculation time to some extent.^[32]^

The current clinical evidence of acupuncture for the treatment of PE is not sufficient. Most of them are low-quality, small-sample results. Therefore, we will use systematic review and meta-analysis to evaluate the efficacy and safety of acupuncture for the treatment of PE. We hope this systematic review will provide more clinical evidence to help doctors and patients make better choices in PE treatment.

It should be noted that there might be limitations in this review. First, the use of language including English and Chinese may induce the bias of the study. Second, different types of acupuncture, acupoints, duration, frequency, the age of patients, and degree of PE may cause high heterogeneity. Third, it is difficult to undertake single or double-blind experiment measures during acupuncture therapy.

## Author contributions

QZ is the guarantor of the article. QZ, HHD, and XHG designed the systematic review. QZ and HHD drafted the protocol and XHG, LW, and MRC revised the manuscript. QZ and HHD will screen the titles, abstracts, keywords of all retrieved records and extract data independently. XHG and LW will assess the ROB independently. LW and MRC will deal with the missing data. HSL and BW will arbitrate any disagreements in the review. All review authors approved the publication of the protocol.

**Conceptualization:** Hai song Li, Bin Wang.

**Data curation:** Qi Zhao, Hengheng Dai.

**Resources:** Minran Cao.

**Software:** Xihao Gong, Lu Wang.
